# Prognostic factors and predictive scores for 6-months mortality of hematopoietic stem cell transplantation recipients admitted to the pediatric intensive care unit

**DOI:** 10.3389/fonc.2023.1161573

**Published:** 2023-09-21

**Authors:** Sarah Schober, Silke Huber, Norbert Braun, Michaela Döring, Peter Lang, Michael Hofbeck, Felix Neunhoeffer, Hanna Renk

**Affiliations:** ^1^ University Children’s Hospital Tuebingen, Department I – General Pediatrics, Hematology/Oncology, Tuebingen, Germany; ^2^ University Children’s Hospital Tuebingen, Department II – Pediatric Cardiology, Pulmonology and Intensive Care Medicine, Tuebingen, Germany

**Keywords:** pediatric, HSCT, PICU, survival, outcome, pSOFA, O-PRISM

## Abstract

**Objective:**

Despite advances in hematopoietic stem cell transplantation (HSCT), a considerable number of pediatric HSCT patients develops post-transplant complications requiring admission to the pediatric intensive care unit (PICU). The objective of this study was to evaluate clinical findings, PICU supportive therapy and outcome as well as predictive factors for 6-months survival after discharge of HSCT patients from PICU.

**Study design:**

This retrospective single-center analysis investigated patient characteristics, microbiological findings, reasons for admission and death of 54 cases accounting for 94 admissions to the PICU of the University Children’s Hospital Tuebingen from 2002 to 2017. We compared clinical characteristics between children with and without 6-months survival after discharge from PICU following HSCT. Finally, we assessed the potential prognostic value of the oncological Pediatric Risk of Mortality Score (O-PRISM), the Pediatric Sequential Organ Failure Assessment Score (pSOFA) and the pRIFLE Criteria for Acute Kidney Injury for 6-months survival using Generalized Estimating Equations (GEE) and Receiver Operating Characteristic curves.

**Results:**

Respiratory insufficiency, gastroenterological problems and sepsis were the most common reasons for PICU admission. Out of 54 patients, 38 (70%) died during or after their last PICU admission, 30% survived for at least six months. When considering only first PICU admissions, we could not determine prognostic factors for 6-months mortality. In contrast, under consideration of all PICU admissions in the GEE model, ventilation (p=0.03) and dialysis (p=0.007) were prognostic factors for 6-months mortality. Furthermore, pSOFA (p=0.04) and O-PRISM (p=0.02) were independent risk factors for 6-months mortality considering all PICU admissions.

**Conclusion:**

Admission of HSCT patients to PICU is still associated with poor outcome and 69% of patients died within 6 months. Need for respiratory support and dialysis are associated with poor outcome. Prediction of 6-months survival is difficult, especially during a first PICU admission. However, on subsequent PICU admissions pSOFA and O-PRISM scores might be useful to predict mortality. These scores should be prospectively evaluated in further studies to verify whether they can identify pediatric HSCT recipients profiting most from transferal to the PICU.

## 1 Introduction

Treatment and outcome of children with cancer have substantially improved during the last two decades. Mortality among hematopoietic stem cell transplantation (HSCT) recipients admitted to pediatric intensive care units (PICUs) has dropped significantly from 91% to about 25% within the last 30 years. However, this is still one of the highest mortality rates among PICU patients (1–4). Survival is determined by different factors such as age, type of HSCT, immune reconstitution, graft versus host disease (GvHD), infections, organ failure and need for organ replacement therapies (1–14).

In stark contrast, the six months survival rate of pediatric patients after HSCT on PICUs has remained relatively unchanged at only 21% to 25% (1, 10).

Advances in PICU patient care including protective ventilation strategies, early and aggressive therapy in sepsis and different options in renal replacement therapy have contributed to the drop in PICU mortality in HSCT patients (1, 5). Other approaches to reduce mortality and morbidity focus on increased pre-PICU symptom surveillance like the Pediatric Early Warning Score (PEWS) (3). Furthermore, changes in oncological treatment such as reduced intensity conditioning, targeted treatment protocols, graft manipulation, patient and donor selection, and advanced supportive therapies contribute to mortality reduction (1, 4, 7).

Around 10% to 40% of all pediatric HSCT recipients are admitted to the PICU at least once (2–5, 9, 15). Besides treatment- or condition-related risk factors, respiratory failure, multiple organ failure and septic shock are major causes for PICU admission (5, 14, 15).

Admission of oncological pediatric patients often raises sensitive questions and ethical issues in parents and healthcare practitioners. Clinical decision-making, e.g. whether a patient should be admitted to PICU at all or intubated or inotropic support should be escalated, is difficult because the outcome after PICU interventions is hard to predict. Furthermore, aggressive interventions need to be balanced against the provision of best end-of-life care through palliative care in the ward or parental support at home. Therefore, data that helps to determine which children may benefit from PICU supportive therapy is crucial to decide the best treatment approach for pediatric HSCT recipients.

Suitable scoring systems for post-HSCT pediatric patients provide a possibility to estimate outcome and the individual mortality risk and may be used to guide clinical decision making. Pediatric Critical Illness Score (PCIS), Pediatric Logistic Organ Dysfunction (PELOD) and the updated version of Pediatric Risk of Mortality (PRISM-3) were of prognostic value for HSCT recipients on PICUs (6, 12, 13, 16), whereas others such as the Pediatric Multiorgan Dysfunction score (PMOD) or the Pediatric Index of Mortality score (PIM-2) showed conflicting data (2, 6, 10, 16). The Oncological Pediatric Risk of Mortality (O-PRISM) score was found to be superior to the Pediatric Risk of Mortality score (PRISM) in a number of studies (1). In PICU patients with acute kidney injury (AKI), the pRIFLE classification (pediatric Risk of renal dysfunction, Injury to the kidney, Failure of kidney function, Loss of kidney function, End-stage kidney diseases) (17, 18) is an important tool to predict hospital mortality and PICU length of stay (19). In 2017, Matics et al. adapted and validated the Sequential Organ Failure Assessment (SOFA) score, which was originally developed for Sepsis outcome, specifically for critically ill children (20). This pediatric SOFA score (pSOFA) had excellent discrimination for in-hospital mortality, with an area under the curve of 0.94 (95% CI, 0.92-0.95). However, to the best of our knowledge, only three studies have applied pSOFA for pediatric HSCT patients to predict PICU mortality and none of them looked at long-term (6-months) survival (21–23). Here, we describe patient characteristics, clinical features, critical care interventions and outcome in a cohort of pediatric HSCT patients, admitted to the PICU of the University Children’s Hospital Tuebingen. This is the first study which explicitly discriminates between first and subsequent PICU admissions to evaluate risk factors for 6-months mortality. The objective is to evaluate the predictive ability of different critical care interventions and scoring systems (O-PRISM, pSOFA and pRIFLE) for the individual mortality risk considering all PICU admissions of a patient by applying a Generalized Estimating Equations (GEE) model. We focus not only on PICU mortality but on long-term (6-months) mortality. This new approach reveals unique insight into long-term prognosis of pediatric HSCT recipients and can support pediatric intensivists and oncologists in clinical decision making.

## 2 Methods

### 2.1 Patient population and study setting

We performed a retrospective, single-center analysis in HSCT patients admitted to the PICU of the University Children’s Hospital Tuebingen during the period from January 2002 to December 2017. This 14-bed PICU cares for critically ill infants and children with up to 920 admissions per year. The main reason for admission is the need for cardiac surgery in about half of all patients, followed by general pediatric surgery and pediatric medical conditions that require intensive care treatment, including patients after HSCT. HSCT is performed by the department of pediatric hematology and oncology at the University Children’s hospital Tuebingen, where about 50 pediatric HSCTs per year are undertaken with a special focus on re-transplantation and haploidentical HSCT. We selected all pediatric HSCT-patients with at least one non-scheduled PICU admission during the observation period and followed them up for any PICU readmission up to two years after HSCT. All PICU admissions due to scheduled post-operative care or interventions such as bronchoscopy, other endoscopies or catheter implantations were excluded from the analysis. The study was approved by the local ethical review board at the University Hospital Tuebingen (project No. 562/2010A) with a waiver of informed consent.

### 2.2 Data acquisition

Demographic, clinical and microbiological data was retrospectively retrieved from patient medical records of the hospital information system (i.s.h. med, SAP). Pediatric patients were included, if they were admitted to the PICU during conditioning or after up to two years after HSCT. Data obtained included age, sex, weight, underlying condition, disease status prior to HSCT, conditioning intensity, type of transplantation and conditioning, transplant-related complications, timing of PICU admission in HSCT, time after HSCT until PICU admission, PICU supportive therapy, number of PICU admissions, duration of PICU stays, reason for PICU admission, 6-months survival, date and cause of death. Microbiological and virological findings were extracted from the hospital laboratory order communication system (LAURIS, nexus/Swisslab). O-PRISM, pSOFA and pRIFLE Scores were determined for the day of PICU admission. Presence of graft-versus host disease (GvHD), thrombotic microangiopathy (TMA) and veno-occlusive disease (VOD) was assessed for every PICU stay and the highest grade of severity was documented. All HSCT patients are routinely monitored for frequent viral pathogens via blood PCR at least once a week. ADV, bacteria and fungi in stool, candida and aspergillus antigen in serum, a swab from the central vascular catheter entrance and a throat swab for bacteria and funguses is performed once a week. BKV in urine is screened once before HSCT. In case of symptoms (e.g. diarrhea, cough), further bacterial and viral diagnostics are performed. All these screening results were evaluated in the analysis presented. The main reason for PICU admission was independently identified by two pediatric oncologists and intensivists after screening of the patient’s history. In case of dissent the two specialists discussed the case and agreed upon one main reason for admission. Cause of death was grouped in accordance with the CLASS system (Classification of death causes after transplantation) (24).

### 2.3 Statistical methods

Patient data was analyzed using Microsoft® Excel, Version 16.12 and IBM® SPSS Statistics Version 22 for Windows. Results are presented as numbers for categorical variables. Normally and not normally distributed quantitative variables are presented as mean ± standard deviation and median (minimum and maximum or interquartile range), respectively. The Kaplan Meier survival analysis was performed using Microsoft® Excel. To determine potential clinically relevant scores and risk factors for 6-months survival, we first applied univariate logistic regression using data from every first PICU admission. Influence of univariate factors with p<0.05 and clinically impactful factors of PICU treatment, known from a previous study (25) were then assessed by generalized estimating equation (GEE) models in order to generally determine the odds ratio of 6-months survival for each risk factor. By adjusting for PICU admission number, the GEE models allow for analysis of repeated measurements or correlated observations, which is the case in multiple PICU admissions of a single patient in our cohort. Every model additionally adjusted for clinically meaningful covariates known from the literature [age group (26), type of transplant, GVHD (27)]. Receiver operating characteristics were constructed and the most appropriate cut-off values for each marker or combination of markers were chosen from the ROC curve by using the point of the curve where the product of the two indices (sensitivity x specificity) is maximum. Cut-off points were used for the calculation of the positive and the negative predictive values.

## 3 Results

**Figure 1:**
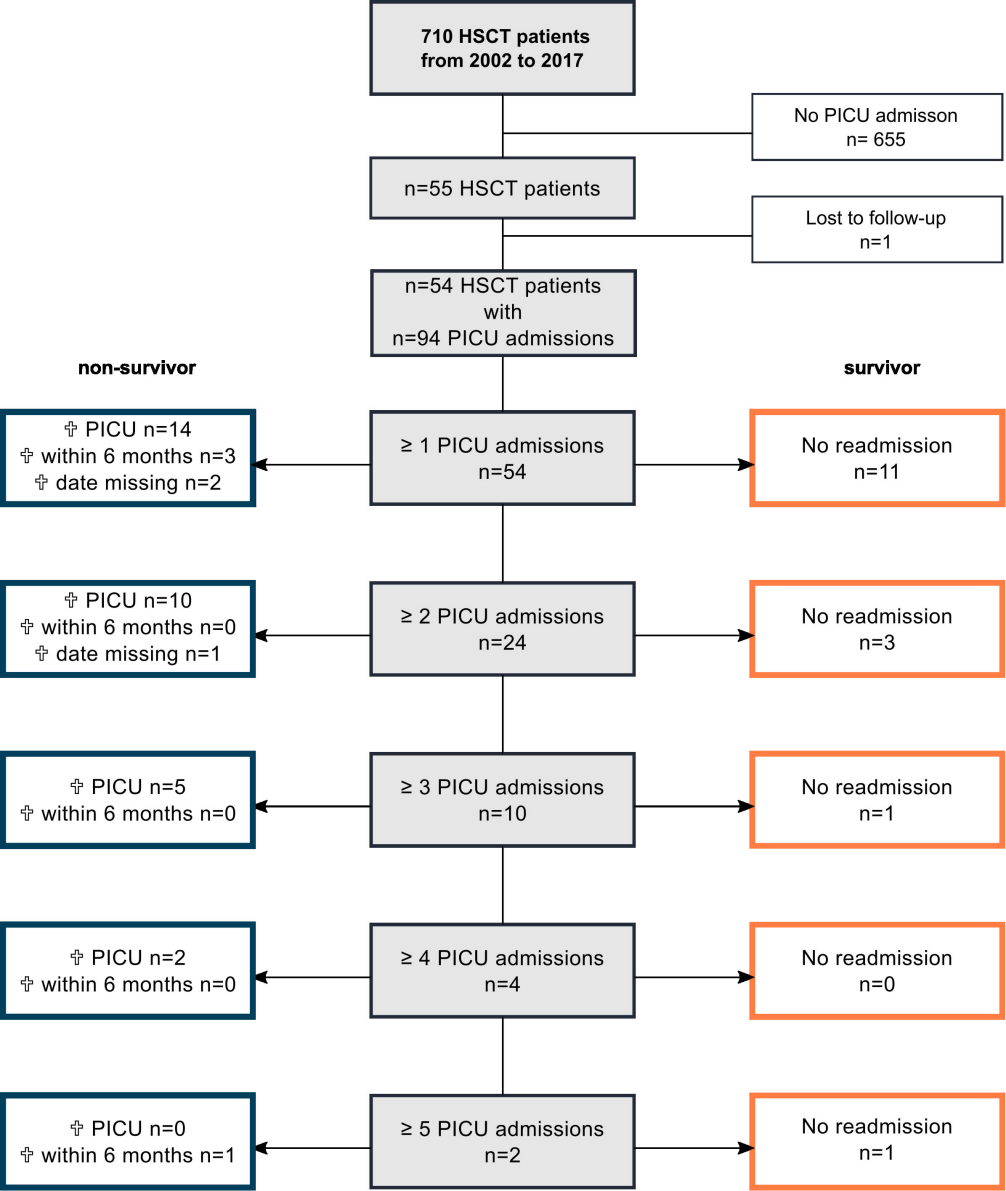
Overview of the study cohort of post-HSCT patients (n=54), their admissions, readmissions and survival. One patient who was lost to follow-up was excluded from the analysis; missing information on 6-months survival in n=3 patients. Analysis requiring 6-months survival data was performed with an n=51.

**Figure 2:**
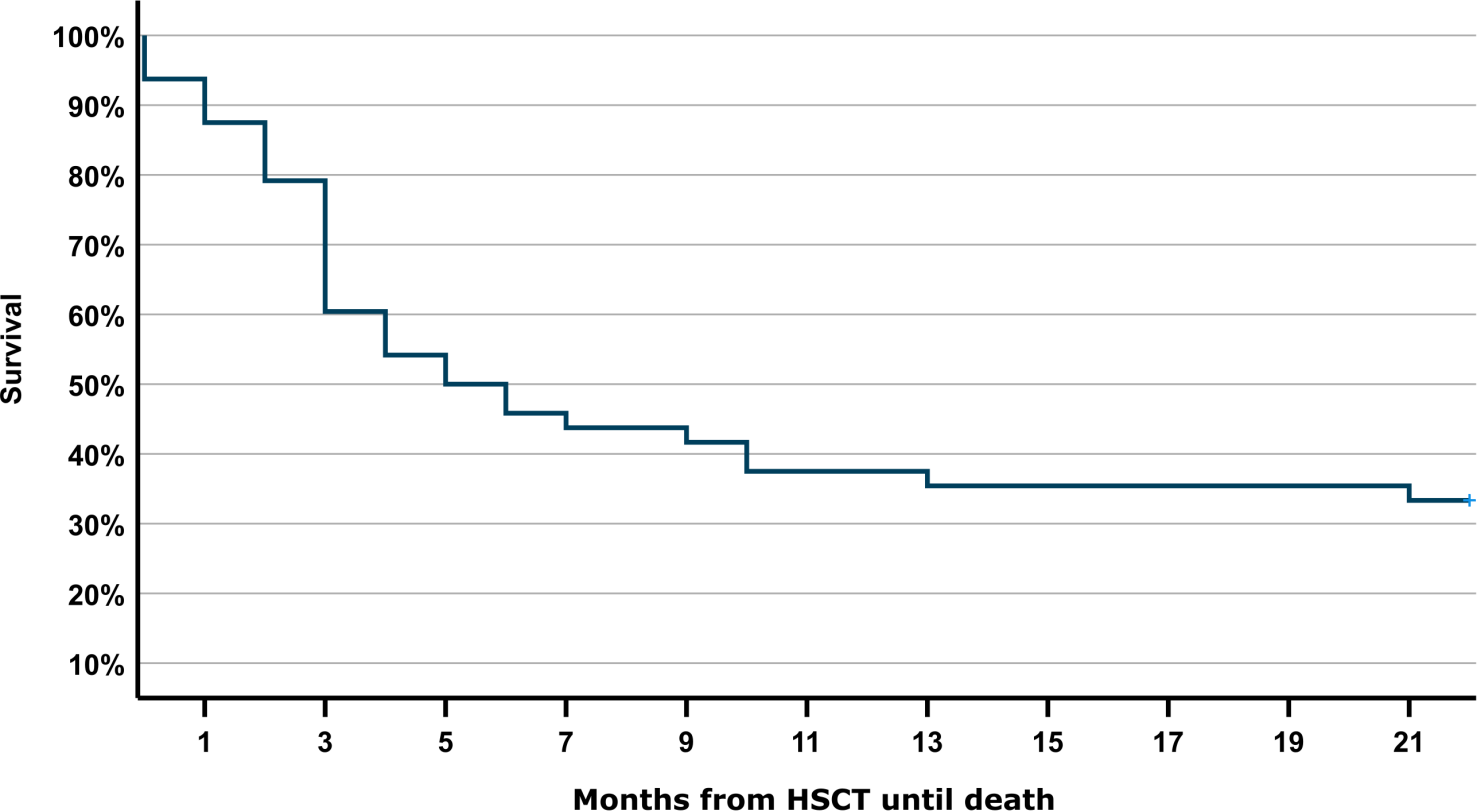
Kaplan-Meier analysis of mortality after pediatric HSCT. Probability of survival (in months) after HSCT and at least one PICU admission. Missing information on 6-months survival in n=3 patients.

A total of 710 patients underwent HSCT during the study period. Of these patients, 54 accounted for a total of 94 admissions to PICU during the study period. 31 boys (57%) and 23 (43%) girls with a median age of 10 years (IQR 5.0-14.8) were admitted to PICU (**Additional Table 1**). 19 patients (35%) died during or after the first PICU admission. Eleven patients (20%) were discharged from PICU and survived and 24 (44%) were readmitted at least once more. Eleven (20%) died during or after the second PICU admission, three (6%) were discharged from PICU after the second admission and survived and ten (19%) were admitted three or more times to PICU. Out of these ten patients only two (4%) survived. In total 31 patients (57%) died during one of their stays on the PICU and 7 died after discharge. The overall 6-months survival rate was 30% (16/54) (**Figure 1**, **Additional Table 1**). The Kaplan-Meier curve demonstrates that almost all non-survivors died during the first six months after HSCT (**Figure 2**).

### 3.1 Reasons for admission to PICU

**Figure 3:**
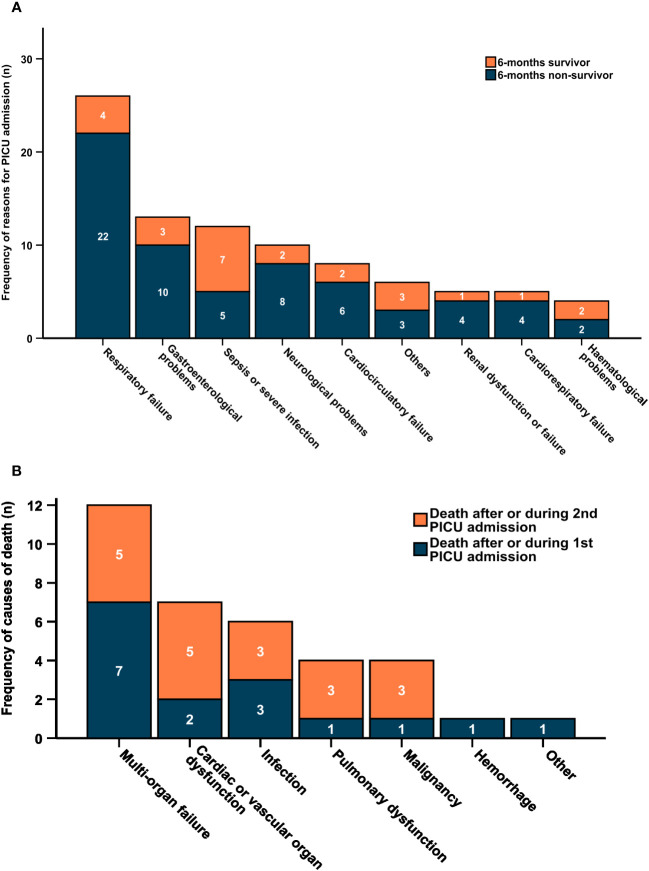
**(A)** Reasons for PICU admission according to 6-months mortality. **(B)** Reasons of death by PICU admission. **(A)** Frequency of main reasons for PICU admission (n=89) after HSCT by 6-months survival. **(B)** Frequency of causes of death (n=35) within 6 months after last PICU admission. Dark blue bars indicate death after or during 1^st^ PICU admission (missing cause of death in n=2), orange bars indicate death after or during 2^nd^ PICU admission.

The most frequent reason for admission to PICU after HSCT was respiratory problems (29.2%) followed by gastroenterological problems including GvHD of the gut or liver, VOD and intestinal bleedings (14.6%) and sepsis (13.5%). The most common reason for PICU admission in 6-months survivors was sepsis, whereas respiratory failure, gastroenterological and neurological problems were most common in 6-months non-survivors. Of note, 6-months non-survivors represented the highest proportion (75-85%) among patients with respiratory failure, gastroenterological and neurological problems, cardiocirculatory failure, renal dysfunction and cardiorespiratory failure as reason for PICU admission. In contrast, sepsis was the main reason for PICU admission in 6-months survivors (28%, **Figure 3A**).

### 3.2 Microbiological and virological findings

Rates of bacterial, viral and fungal organisms per admission group were detected by routine screening on each PICU admission (or up to one week before) (**Additional Figures 1A–C**, **Additional Table 2**). Cumulative rates and rates of each detected organism are displayed for each PICU admission without readmission, with readmission or non-survival during or after the respective PICU stay. Overall, bacterial isolates were detected most frequently when no further PICU admission was required. Enterococci and coagulase-negative Staphylococci accounted for about 50% of detected organisms when readmission was required or only one admission was necessary. On the contrary, in non-survivors *Clostridioides difficile* and *Pseudomonas/Stenotrophomonas spp* were isolated in about 50% of admissions (**Additional Figure 1A**). In non-survivors during or after PICU admission *Adenovirus* (ADV) was found most frequently, followed by *BK-Virus* (BKV) and *Human Herpesvirus 6* (HHV 6). In the case of readmission to PICU a similar distribution of viruses was found. However, ADV was less frequent. In patients without readmission, *BK-Virus* was most commonly isolated (**Additional Figure 1B**). In contrast to the decreasing rate of bacterial isolates with readmission and non-survival, fungal isolates were almost twice as common in non-survivors as in patients who required no further readmission to PICU. Distribution of fungal isolates was clearly dominated by *Candida* and *Aspergillus spp* in all admission groups (**Additional Figure 1C**).

### 3.3 Cause of death

35 out of 51 patients, for whom data on 6-months survival is available, died. Multi-organ failure was the most common cause of death (34%) followed by cardiac or vascular organ dysfunction (20%) and infections (17%). This distribution is rather similar in patients dying during or after the first or second PICU admission. In total, just 4 (11%) patients died due to the underlying malignancy/relapse (relapse-related mortality). The relapse-related mortality was more relevant after or during the second PICU admission. However, with 89% the transplant-related (non-relapse-related) mortality was by far more relevant in the presented cohort (**Figure 3B**).

### 3.4 Comparison of 6-months survivors and 6-months non-survivors

All transplant-related details for 51 patients in which data on 6-months mortality was available are listed in **Table 1**. ALL (n=15), primary immunodeficiency (n=7) and solid tumors (n=7) were the most frequent underlying diseases. Six (12%) patients had undergone autologous transplantation. 45 (88%) had received allogeneic HSCT, including 20 (39%) haploidentical HSCT. The median period until first PICU admission after HSCT was 50 days with a wide range from -15 to 378 days. Median length of PICU stay was 6 days. In regards to HSCT related side effects GvHD was present in 26 (51%) patients, thrombotic microangiopathy in 10 patients and VOD in 9 patients.

**Table 1:** Patient, disease, HSCT and PICU treatment characteristics of 6-months survivors and 6-months non-survivors (n=51*).

	6-months survivor (n=16)	6-months non-survivor (n=35)	Total (n=51*)
Patient characteristics			
**Sex m/f**	10/6	21/14	31/20
**Median age in years; [IQR]**	10 [5-16]	9 [4-15]	10 [5-15]
**Weight (kg) before HSCT; mean ± SD**	39 ± 23	35 ± 23	36 ± 23
**Weight (kg) on PICU admission; mean ± SD**	37 ± 22	34 ± 21	34 ± 21
Underlying disease			
**ALL**	5	10	15
**AML**	1	4	5
**CML**	2	0	2
**JMML**	0	2	2
**MDS**	0	3	3
**Lymphoma**	1	1	2
**Solid tumor**	3	4	7
**PID**	2	5	7
**AA**	1	0	1
**WAS**	0	1	1
**Others**	1	5	6
Disease status prior to HSCT			
**Complete remission** **Active malignancy** **Non-malignant disease**	8 2 4	11 10 11	19 12 15
Received therapy before HSCT and conditioning			
**No conditioning** **Myeloablative** **Reduced intensity**	1 11 3	0 28 5	1 39 8
Donor type			
**Autologous**	3	3	6
**Allogeneic^1^, total**	13	32	45
**- haploidentical**	6	14	20
**- matched related donor**	0	6	6
**- matched unrelated donor**	6	10	16
**- cord blood**	0	2	2
Complications			
**TMA**	3	7	10
**VOD**	2	7	9
**GvHD (any)**	7	19	26
**GvHD gut**	3	13	26
**GvHD liver**	2	5	7
**GvHD skin**	5	14	19
**Occurrence of aGVHD^2^**	7	18	25
**I-II aGVHD**	3	7	11
**III-IV aGVHD**	3	9	12
**Occurrence of cGVHD^2^**	2	2	4
**Mild cGVHD**	1	0	1
**Moderate cGVHD**	0	1	1
**Severe cGVHD**	1	1	2
First PICU admission and treatment			
**PICU admission after 1^st^ HSCT**	11	29	40
**PICU admission after 2^nd^ HSCT**	5	6	11
Timepoint of first PICU admission			
**Conditioning** **Pre-engraftment** **Post-engraftment**	1 4 11	4 6 24	5 10 35
**Days after HSCT until first PICU admission; median, [range]**	29 [-15; 246]	51 [-10; 378]	50 [-15;378]
**Length of first PICU admission;** **median days, [range]**	4 [1; 34]	6 [1; 39]	6 [1;39]
**MOF**	13	32	45
**Ventilation**	6	16	22
**Circulatory support**	8	16	24
**Dialysis**	1	11	12
**ECMO**	0	3	3
**pSOFA; median [range]**	9 [3; 13]	10 [5; 17]	10 [3;17]
**O-PRISM; median [range]**	22 [7; 39]	26 [10; 48]	26 [7;48]
**pRIFLE; median [range]**	2 [0; 3]	2 [0; 4]	2 [0;4]
Cause of death			
**Relapse-related mortality** **Non-relapse-related mortality**	0	4 31	35

IQR, interquartile range; SD, standard deviation; TMA, thrombotic microangiopathy; GvHD, graft versus host disease; VOD, veno-occlusive disease; MOF, multi-organ failure; ECMO, extracorporeal membrane oxygenation; pSOFA, pediatric Sequential Organ Failure Assessment Score; O-PRISM, Oncological Pediatric Risk of Mortality Score; pRIFLE, pediatric Risk of renal dysfunction, Injury to the kidney, Failure of kidney function, Loss of kidney function, End-stage kidney diseases. Patient characteristics, underlying disease, disease status, received therapy and donor type are displayed for every first PICU admission. Scores were determined for the day of PICU admission and medians are shown for every patient’s first PICU admission. GvHD, TMA and VOD were counted if present during any PICU stay and the highest grade of severity was documented. There is missing information on 6-months survival in 3 patients (*), therefore total n=51. ^1^in one patient, only allogeneic but not special type is known. Missing data on disease status prior to HSCT in 5 patients, in received therapy in 3 patients and timepoint of first PICU admission is unknown in one patient. ^2^includes patients with the combination of acute and chronic GVHD.

All patients with JMML (juvenile myelomonocytic leukemia), MDS (myelodysplastic syndrome) and WAS (Wiskott-Aldrich-syndrome) as well as the vast majority of patients with primary immunodeficiency (5/7) and AML (4/5) died during or within 6 months after PICU admission. 83% of all patients being transplanted with an active malignancy died (details see **Table 1**).

Three patients underwent Extracorporeal Membrane Oxygenation (ECMO) on their first PICU admission, but all died. It is worth to mention one additional patient, who was readmitted twice to the PICU beyond the pre-defined observation period of this study (>3 years after HSCT), who underwent ECMO and survived.

### 3.5 Prediction of 6-months mortality

**Figure 4:**
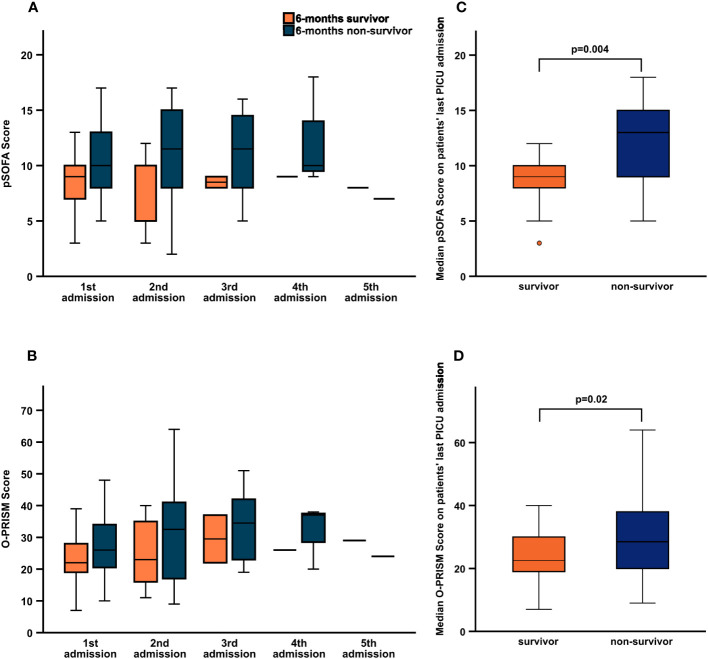
Comparison of pSOFA and O-PRISM score between 6-months survivors and 6-months non-survivors. Median pSOFA **(A)** and O-PRISM **(B)** score distributed by number of PICU admission and median pSOFA **(C)** and O-PRISM **(D)** score of patients' last PICU admission for 6-months survivors (orange) and non-survivors (blue). For numbers (median, range) see **Additional Table 3**.

pSOFA and O-PRISM scores increased with number of PICU admission, although less data could be evaluated due to a decreasing number of patients for every additional readmission (**Figures 4A, B**). Differences in median scores between 6-months survivors and non-survivors could not be detected when only considering all first PICU admissions of our cohort, but in all patients’ last admissions (**Table 1**, **Figures 4C, D**, **Additional Table 3**). Furthermore, univariate logistic regression analysis did not reveal any of the disease scores or critical care interventions as predictive for 6-months mortality in this patient subset (**Table 2**). In contrast, consideration of all admissions to the PICU of a single patient confirmed pSOFA and O-PRISM as well as respiratory support and dialysis as predictive factors for 6-months mortality (**Table 2**). Due to the longitudinal data structure with different numbers of admissions for every patient, generalized estimating equations were applied and models corrected for admission number, age-group, type of transplant and GVHD. Overall, pSOFA and O-PRISM were associated with 6-months mortality with an adjusted OR of 1.04 (95% CI 1.00-1.07, p=0.04, QIC 113.48) and 1.01 (95% CI 1.00-1.02, p=0.02, QIC 114.44). When examining the different PICU interventions, respiratory support and dialysis increased the risk for 6-months mortality with an adjusted OR of 1.21 (95% CI 1.02-1.44, p=0.03, QIC 113.60) and 1.67 (95% CI 1.15-2.44, p=0.007, QIC 106.67), respectively. This was not true for cardiocirculatory support (**Table 2**).

**Table 2:** Relationship between main variables and 6 months mortality after PICU discharge using logistic regression analysis and Generalized estimating equations (GEE) models.

		Univariate logistic regression		Generalized estimating equations		
Variable	n	Every 1^st^ PICU admission OR (95% CI)	p-value	Adjusted OR (95% CI)	p-value	QIC
**pSOFA**	50	1.12 (0.94-1.35)	0.21	1.04 (1.00 – 1.07)	0.04	113.48
**O-PRISM**	50	1.05 (0.98-1.13)	0.15	1.01 (1.00 – 1.02)	0.02	114.44
**Respiratory support**	50	1.77 (0.53-5.92)	0.36	1.21 (1.02 – 1.44)	0.03	113.60
**Cardiocirculatory support**	50	0.89 (0.27-2.92)	0.85	1.07 (0.95 – 1.20)	0.29	116.32
Dialysis*	48	7.17 (0.84-61.46)	0.07	1.67 (1.15 – 2.44)	0.007	106.67

Univariate logistic regression on every first PICU admission of each patient and Generalized estimating equations model of n=85 PICU admissions (n=83 for analysis of dialysis). Each model was adjusted for the following covariates: number of admissions and patient-specific confounders (age group, type of transplant and GVHD). *Dialysis was not adjusted for type of transplant due to multicollinearity. Respiratory support includes invasive ventilation or non-invasive ventilation. P-value for adjusted OR. OR: odds ratio; CI: confidence interval; QIC: Quasi-likelihood under the independence model criterion (QIC) for choosing the best correlation structure.

Receiver operating characteristic curves (ROC) analysis of both scores was performed separately for every PICU admission in order to identify an optimal cut-off for prediction of 6-months mortality (**Additional Figures 2A, B**). During the second PICU admission sensitivity and PPV of pSOFA, was highest (94.44%, 95% CI 72.7-99.9, PPV 89.5%, 95% CI 74.3-96.2) with an area under the ROC curve (AUC) of 0.78 and cut-off of 6.0, O-PRISM showed a maximum sensitivity of 75.0% (95% CI 34.9-96.8) and PPV 85.7% (95% CI 58.6-96.2) with an AUC of 0.59 and a cut-off of 24.5 during the third admission (**Table 3**). No single optimal cut-off could be identified for both scores.

**Table 3:** Predictability of pSOFA and O-PRISM during 1^st^ - 4^th^ PICU admission.

Variable	n	AUC	Cut-off	Sensitivity % (95% CI)	Specificity % (95% CI)	PPV % (95% CI)	NPV % (95% CI)	Accuracy % (95% CI)
**pSOFA** **1^st^ PICU admission**	49	0.62	9.5	53.12 (34.7-70.9)	58.8 (32.9-81.6)	70.8 (55.8-82.4)	40.0 (27.9-53.4)	55.1 (40.2 – 69.3)
**pSOFA** **2^nd^ PICU** **admission**	23	0.78	6.0	94.44 (72.7-99.9)	60.0 (14.7-94.7)	89.5 (74.3-96.2)	75.0 (28.2-95.8)	87.0 (66.4-97.2)
**pSOFA** **3^rd^ PICU admission**	10	0.69	8.5	62.5 (24.5-91.5)	50.0 (1.26-98.7)	83.3 (53.1-95.7)	25.0 (6.0-63.4)	60.0 (26.2-87.8)
**pSOFA** **4^th^ PICU admission**	4	0.83	9.5	66.7 (9.4-99.2)	100.0 (2.5-100.0)	100.0	50.0 (16.8-83.2)	75.0 (19.4-99.4)
**pSOFA every last PICU admission**	50	0.76	9.5	71.4 (53.7-85.4)	60.0 (32.3-83.7)	80.7 (68.4-88.9)	47.4 (31.6-63.7)	68.0 (53.3-80.5)
**O-PRISM** **1^st^ PICU admission**	49	0.63	22.5	71.9 (53.3-86.3)	58.8 (32.9-81.6)	76.7 (64.1-85.8)	52.6 (36.0-68.7)	67.4 (52.5-80.1)
**O-PRISM** **2^nd^ PICU** **Admission**	23	0.64	25.0	61.1 (35.8-82.7)	60.0 (14.7-94.7)	84.6 (63.9-94.5)	30.0 (14.6-51.8)	60.9 (38.5-80.3)
**O-PRISM** **3^rd^ PICU admission**	10	0.59	24.5	75.0 (34.9-96.8)	50.0 (1.3-98.7)	85.7 (58.6-96.2)	33.3 (7.4-75.8)	70.0 (34.8-93.3)
**O-PRISM** **4^th^ PICU admission**	4	0.67	23.0	66.7 (9.43-99.2)	0.0 (0.0-97.5)	66.7 (47.3-81.7)	0	50.0 (6.8-93.2)
**O-PRISM every last PICU admission**	50	0.71	23.5	68.6 (50.7-83.2)	60.0 (32.3-83.7)	80.0 (67.4-88.6)	45.0 (30.1-60.8)	66.0 (51.2-78.8)

AUC and Cut-off for pSOFA and O-PRISM on 1^st^ – 4^th^ admission N for every number of PICU admission is given, missing data on scores in n=1 patient. Sensitivity, specificity, positive and negative predictive values are expressed as percentages. Confidence intervals for sensitivity and specificity are “exact” Clopper-Pearson confidence intervals. Confidence intervals for the predictive values are standard logit confidence intervals. AUC, area under the curve; NPV, negative predictive value; PPV, positive predictive value.

## 4 Discussion

During the last decades there has been remarkable progress in pediatric oncology with increasing life expectancy and improving prognosis in many areas. However, the prognosis of children that are admitted to PICU after HSCT is still quite poor. Here, we describe a pediatric HSCT cohort of 54 children admitted to the PICU in more detail and analyze potential prognostic factors for 6-months mortality.

In line with other contemporary studies (5, 28), PICU mortality of our cohort was 57% (31/54) and 6-months mortality was 65% (35/54). This means an additional 6% of patients died within 180 days after their last PICU discharge. Compared to a study that was performed at our hospital 18 years ago (25), 6-months survival rate has increased from 23% to 30%. With five times the observation period in the current study (3 vs. 15 years), the number of PICU patients only doubled (23 vs. 54 patients) compared to the previous study. However, the average number of PICU admissions decreased from 9 PICU admissions per year in the former study (26 admissions in 23 patients) compared to 6 PICU admissions per year (94 admissions in 54 patients). This result could be related to a different PICU admission strategy at earlier timepoints, a shorter time per admission to the PICU in line with the availability of moving patients between PICU and HSCT intermediate care wards. Consistent with the patient structure in previous studies, the most common underlying disease for HSCT was ALL (6, 25). Solid tumors and primary immunodeficiency disorders (PID) represented the second largest group, which might be due to improved diagnostics and the expertise in our center. Of note, haploidentical transplantation represented the most frequent transplant mode followed by matched unrelated donor in the current study. 18 years ago haploidentical HSCT was the most common type of transplantation as well (25).

The most important cause for PICU admission in our cohort was respiratory failure, followed by gastrointestinal problems and sepsis. Importantly, respiratory failure or the combination of respiratory and cardiocirculatory failure as well as renal dysfunction or failure and neurological problems were present in the vast majority (75-85%) of patients that died within 6-months after PICU admission. On the contrary, 6-months survivors accounted for the majority of patients admitted with sepsis. The striking role of respiratory failure as the main reason for admission to PICU in our study is well known from HSCT and non-HSCT hemato-oncologic patients (5, 8). However, compared to the past, when almost all PICU admissions resulted in mechanical ventilation, less than half of the HSCT recipients (22/51) needed mechanical ventilation and half of all patients needed circulatory support in our current cohort. In this context the role of non-invasive ventilation (NIV) strategies in HSCT patients is still under investigation with several conflicting study results. On the one hand, invasive mechanical ventilation considerably increases the risk of mortality (3, 4, 13, 14). Early use of NIV to prevent intubation might be a promising option and is associated with lower mortality rate in some studies (3, 14). In other analysis non-invasive ventilation use pre-intubation was associated with increased mortality in pediatric HSCT patients (28, 29). Further studies are required to evaluate NIV in HSCT patients. Although not systematically assessed, the distribution and cumulative rate of bacterial, viral and fungal isolates among patients without and with PICU readmission compared to non-survivors revealed some interesting insights. Gram-negative rods, *Pseudomonas spp* and *Clostridioides difficile* were overrepresented in 6-months non-survivors. Furthermore, among viral isolates ADV clearly dominated in 6-months non-survivors. This is consistent with the current literature stating the highest infection-associated mortality rate of 42% if ADV is present at admission (compared to a total mortality rate of 16.2% of all PICU admissions in the same cohort) (3). The frequency of fungal isolates was highest in six months non-survivors and dominated by *Candida spp* and *Aspergillus* spp.

In order to gain a better understanding of why patients died early during or after the first PICU admission compared to later during or after a second or further PICU admissions, we analyzed the frequency of causes of death. In general the relapse-related mortality (RRM) was rather low (4/51), however the non-relapse but transplant-related mortality was considerable high with 31/51. Multi-organ failure and cardiac or vascular organ dysfunction were given reasons in more than half of all deaths, followed by infections. These findings are consistent with other reports from the literature (11).

### 4.1 Prediction of outcome and value of pSOFA

Overall, when considering every first PICU admission, no difference of demographic features, type of treatment, frequency of GvHD, presence of MOF and need for supportive therapy was found between 6-months survivors and 6-months non-survivors. Only the frequency of dialysis, reflecting renal failure seemed to be more frequent in six months non-survivors. Sustained renal failure and failed negative fluid management have already been identified as significant mortality risk factors in the previous study in our center (25). Of note, 3 patients received PICU supportive therapy via ECMO on their first admission, all of whom died. This is supported by a high PICU mortality rate of 77.8% for HSCT patients on ECMO given in the literature (4). On the other hand, there are a few case reports of successful ECMO treatment in non-malignant HSCT patients (1). Thus, it is debatable whether ECMO-therapy should be offered to HSCT patients due to unfavorable prognosis. To that end, an international and multidisciplinary consensus statement on the use of ECMO in children receiving HSCT has been published only recently as a clinical decision support tool in these difficult situations (30). In order to find a suitable prognostic tool to predict 6-months survival, we assessed the predictive ability of O-PRISM and pSOFA as well as the need for PICU supportive therapy for 6-months survival within our cohort. To account for multiple PICU admissions of each patient, we used GEE models. The adjusted odds ratio confirmed pSOFA and O-PRISM as prognostic factors for 6-months survival, although cut-offs determined by ROC curves did not perform well. A recent study including 110 pediatric oncology patients found a cut-off value of pSOFA of ≥ 8 for discriminating mortality (22). Furthermore, serial evaluation of SOFA score during the first few days after PICU admission was a good predictor of prognosis and correlated with mortality in pediatric oncology patients requiring mechanical ventilation (31). This supports our finding, that pSOFA is useful in pediatric HSCT patients requiring repetitive PICU admissions. However, here we could not determine a clear cut-off of pSOFA or O-PRISM to decide which children may benefit from repetitive PICU admissions or escalation of therapy as opposed to supportive or palliative care outside the PICU.

Interestingly, when we specifically assessed all PICU admissions of each patient using GEE models, we also found a significantly higher risk of 6-months mortality in patients undergoing dialysis or with the need for ventilatory support. Therefore, we hypothesize that long-term need for PICU supportive therapy, in particular mechanical ventilation and dialysis are predictors of poor outcome.

The present study has some limitations. First, this study is limited by its retrospective, single-center design with a rather small cohort size. Transfer and admission criteria of HSCT recipients to a PICU may differ between hospitals and countries and thus our results may not be applicable in different settings. Second, changes in clinical patient care or criteria for PICU admission during the study period might have an impact on the presented results. Third, the retrospective evaluation of predictive scoring system is always dependent on the quality of clinical data. It should be kept in mind that regardless of which score is applied, they anticipate population mortality risk and not individual prognosis. Additionally, pSOFA focuses on organ malfunction in sepsis including thrombocyte count and hyperbilirubinemia. These two factors are often pathological in post HSCT patients as thrombocytopenia might be present due to delayed hematopoietic reconstitution and hyperbilirubinemia based on transient VOD or drug toxicity. Considering these causes not being associated with high mortality, thrombocytopenia and hyperbilirubinemia seem not to be adequate parameters to predict outcome in HSCT patients. Furthermore, O-PRISM was established for the presented target group of children requiring ICU treatment following HSCT. The score and its parameters are based on a retrospective analysis in a single center setting and a prospective evaluation in the same center (32, 33) and includes the standard PRISM score and three additional variables (CRP, GVHD and hemorrhage). As with pSOFA, PRISM also includes liver function presented by PTT and bilirubin which might not be suitable parameters in HSCT patients. Finally, we did not take into consideration quality of life and disease burden in this study. Nevertheless, our analysis provides an important approach for a further prospective assessment of the predictive ability of the pSOFA and O-PRISM score, including a larger number of pediatric oncology patients from multiple centers with more than one PICU admission.

In conclusion, admission of HSCT patients to PICU is still associated with poor outcome since 65% of patients died within six months. In particular, mechanical ventilation and dialysis seem to be associated with poor outcome. In contrast to the first PICU admission of HSCT patients, pSOFA and O-PRISM might be of particular predictive value in repetitive PICU admissions. However, further research is certainly required to disentangle whether pSOFA and O-PRISM can predict which patients benefit most from continued PICU supportive therapy and whether these scores can inform end of life decisions.

## Data availability statement

The data analyzed in this study is subject to the following licenses/restrictions: Informed consent was not obtained to make single pseudonymized participant data publicly available. Requests to access these datasets should be directed to hanna.renk@med.uni-tuebingen.de.

## Ethics statement

The study was approved by the local ethical review board at the University Hospital Tuebingen (project No. 562/2010A) with a waiver of informed consent. The studies were conducted in accordance with the local legislation and institutional requirements. Written informed consent for participation was not required from the participants or the participants' legal guardians/next of kin in accordance with the national legislation and institutional requirements.

## Author contributions

FN, HR, and SS are responsible for the conception and design of the study. SS and HR performed the statistical analysis and drafted the manuscript. SH and NB have made substantial contributions to the acquisition of the data. FN, MD, PL, and MH revised this manuscript critically for important intellectual content. All authors finally approved this version of the manuscript for submissions. The authors agree to be accountable for all aspects of the work in ensuring that questions related to the accuracy or integrity of any part of the work are appropriately investigated and resolved. All authors confirm that they had full access to all the data in the study and accept responsibility for the decision to submit for publication. All authors contributed to the article and approved the submitted version.
