# Supplementary material for: Prognostic factors and predictive scores for 6-months mortality of hematopoietic stem cell transplantation recipients admitted to the pediatric intensive care unit
Source: Front Oncol. 2023 Sep 21;13:1161573. doi: 10.3389/fonc.2023.1161573 (PMC10552149; doi:10.3389/fonc.2023.1161573)
Supplement: Supplementary file 1 [file DataSheet_1.pdf]

frontiers

Additional file

Prognostic factors and predictive scores for 6-months mortality of hematopoietic stem cell transplantation recipients admitted to the pediatric intensive care unit

Sarah Schober[1*], Silke Huber[2], Norbert Braun[2], Michaela Döring[1], Peter Lang[1], Michael Hofbeck[2], Felix Neunhoeffer[2], Hanna Renk[1,2]

[1]University Children's Hospital Tuebingen, Department I – General Pediatrics, Hematology/Oncology, Hoppe-Seyler-Str.1, Tuebingen, Germany

[2]University Children's Hospital Tuebingen, Department II – Pediatric Cardiology, Pulmonology and Intensive Care Medicine, Hoppe-Seyler-Str.1, Tuebingen, Germany

I. Tables

Additional Table 1. Demographic data and outcome of the cohort

Description	N (%)
PICU admissions after HSCT	94
Patients admitted to PICU after HSCT	54
Median age (range)	10 (5-14.8)
Male	31 (57.4)
Female	23 (42.6)
Patients with 1 PICU admission<br>Patients with 2 PICU admissions<br>Patients with 3 PICU admissions<br>Patients with 4 PICU admissions<br>Patients with 5 PICU admissions	30 (55.5)<br>14 (25.9)<br>6 (11.1)<br>2 (3.7)<br>2 (3.7)
1st PICU admission survivor<br>1st PICU admission non-survivor<br>(On 1st PICU and after 1st PICU admission)<br>1st PICU readmission<br>1st PICU survivors without readmission	35 (64.8)<br>19 (35.2)<br><br>24 (44.4)<br>11 (20.4)
Deaths on PICU	31 (57%)

Patients 6-months survivor*	16 (29.6)
Patients 6-months death*	35 (64.8)

Legend Additional Table 1. Frequencies are numbers and (percentages). *missing information on 6-months survival in n=3 patients.

Additional Table 2. Rate of bacterial, viral and fungal isolates per admission and group

	No readmission (n=16 admissions)	Readmission (n=40 admissions)	Non-survivor (n=38 admissions)
**Bacterial isolates per admission and group**	**0.63**	**0.45**	**0.42**
Coagulase-negative Staphylococci	0.19	0.20	0.03
Enterococcus spp. (S)	0.13	0.03	0.03
Vancomycin-resistant Enterococci	0.06	0.05	0.03
Gram-neg. Rods (S)	0.13	0.08	0.11
Clostridoides difficile	0.00	0.05	0.06
Pseudomonas and Stenotrophomonas spp.	0.06	0.03	0.14
Others (Lactobacillus, Mycoplasma, Streptococci)	0.06	0.03	0.03
**Viral isolates per admission and group**	**0.94**	**1.28**	**1.22**
ADV	0.06	0.15	0.39
BKV	0.44	0.25	0.22
CMV	0.06	0.23	0.14
EBV	0.00	0.10	0.08
JCV	0.06	0.08	0.03
Norovirus	0.00	0.08	0.06
Rotavirus	0.06	0.03	0.00
RSV	0.00	0.03	0.03
HSV	0.06	0.05	0.00
HHV6	0.13	0.15	0.22
Parainfluenza	0.00	0.15	0.00
Others (HPV, HBV, ParvoB19)	0.06	0.00	0.06
**Fungal isolates per admission and group**	**0.44**	**0.63**	**0.81**
Aspergillus spp.	0.13	0.18	0.33
Candida spp.	0.25	0.33	0.39
Fusarium spp.	0.00	0.03	0.03
Trichosporon mucoides	0.00	0.00	0.03
Saccharomyces spp.	0.00	0.08	0.03
Blastoschizomyces spp.	0.06	0.03	0.00

Legend Additional Table 2. VRE:Vancomycin-resistant Enterococcus. ADV: Adenovirus, BKV: BK Virus, CMV: Cytomegalovirus, EBV: Epstein Barr Virus, JCV: JC Virus, RSV: Respiratory Syncitial Virus, HSV: Herpes Simplex Virus, HHV6: Human Herpesvirus 6, HPV: Human Papillomavirus, HBV: Hepatitis B Virus, ParvoB19: Parvovirus B 19, S – sensitive.

Additional Table 3. Median pSOFA and O-PRISM score distributed by number of PICU admission for 6-month survivors and non-survivors.

**PICU Admission**	**6-months survival post PICU discharge**			**6-months non-survival post PICU discharge**		
	Median pSOFA (IQR)	Median O-PRISM (IQR)	Number of admissions (n)	Median pSOFA (IQR)	Median O-PRISM (IQR)	Number of admissions (n)
1	9 (7-10)	22 (109-28	17	10 (8-13)	26 (21-34)	32
2	5 (5-10)	23 (16-35)	5	12 (8-15)	33 (17-41)	18
3	9 (8-9)	30 (22-37)	2	12 (8-15)	35 (23-42)	8
4	9 (n.a)	26 (n.a.)	1	10 (9-18)	37 (20-38)	3
5	8 (n.a.)	29 (n.a.)	1	7 (n.a..)	24 (n.a.)	1
Total	9 (8-10)	22 (20-30)	15*	13 (9-15)	33 (23-41)	35

Legend Additional Table 3. Medians and IQR are given for the respective PICU admission. Data on 6-month survival and non-survival refer to each PICU admission. Total Medians refer to scores at every patient's last PICU admission. *missing score in one 6-month survivor.

II. Figures

Additional Figure 1 Cumulative and pathogen specific rate of bacterial (A), viral (B), and fungal (C) isolates per admission and group.

Legend Additional Figure 1. VRE:Vancomycin-resistant Enterococcus. ADV: Adenovirus, BKV: BK Virus, CMV: Cytomegalovirus, EBV: Epstein Barr Virus, JCV: JC Virus, RSV: Respiratory Syncitial Virus, HSV: Herpes Simplex Virus, HHV6: Human Herpesvirus 6.

(A)

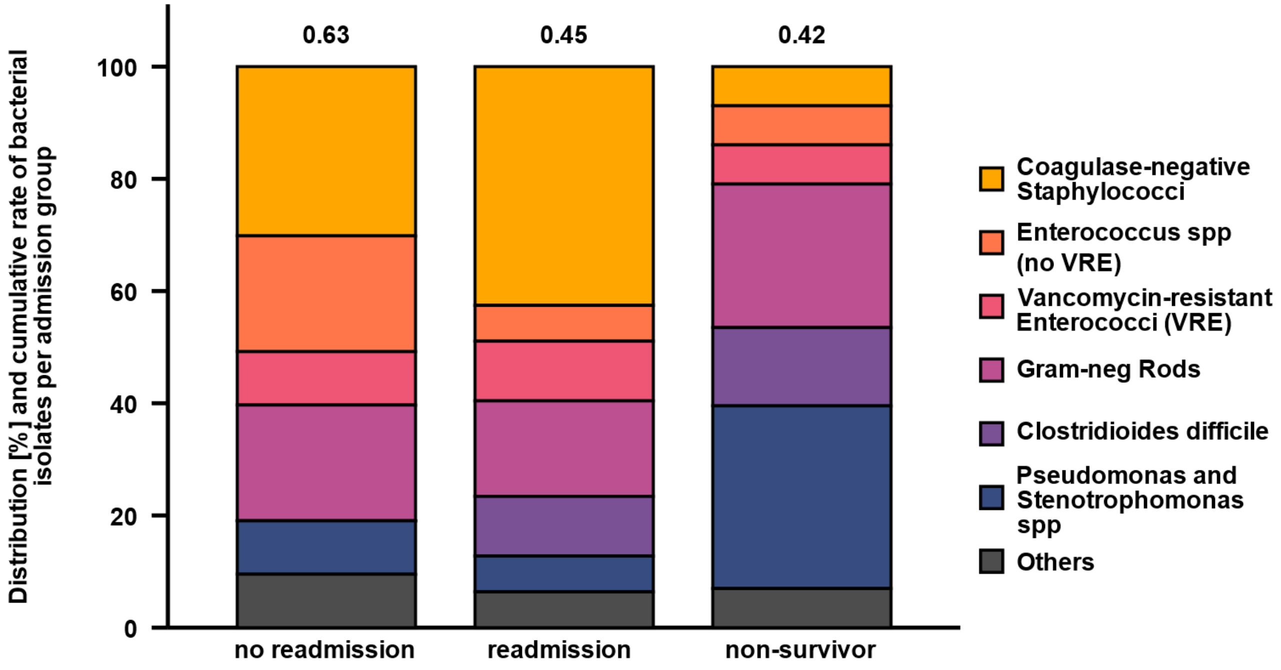

(B)

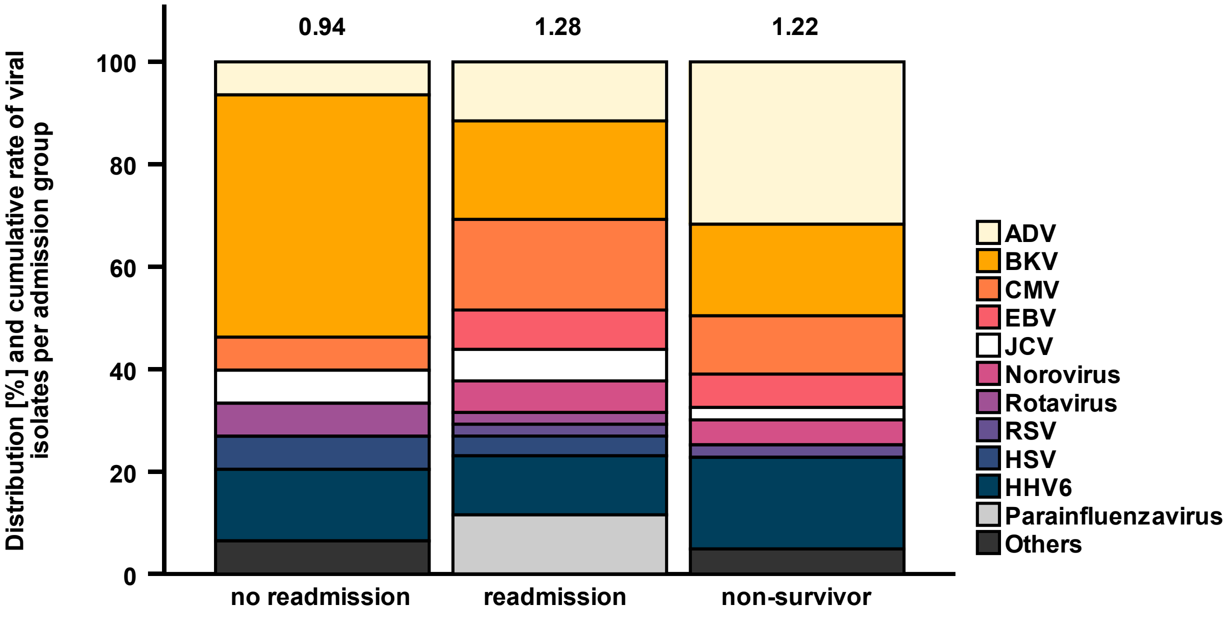
0.94
1.28
1.22
Distribution [%] and cumulative rate of viral isolates per admission group
100
80
60
40
20
0
no readmission
readmission
non-survivor
ADV
BKV
CMV
EBV
JCV
Norovirus
Rotavirus
RSV
HSV
HHV6
Parainfluenzavirus
Others

(C)

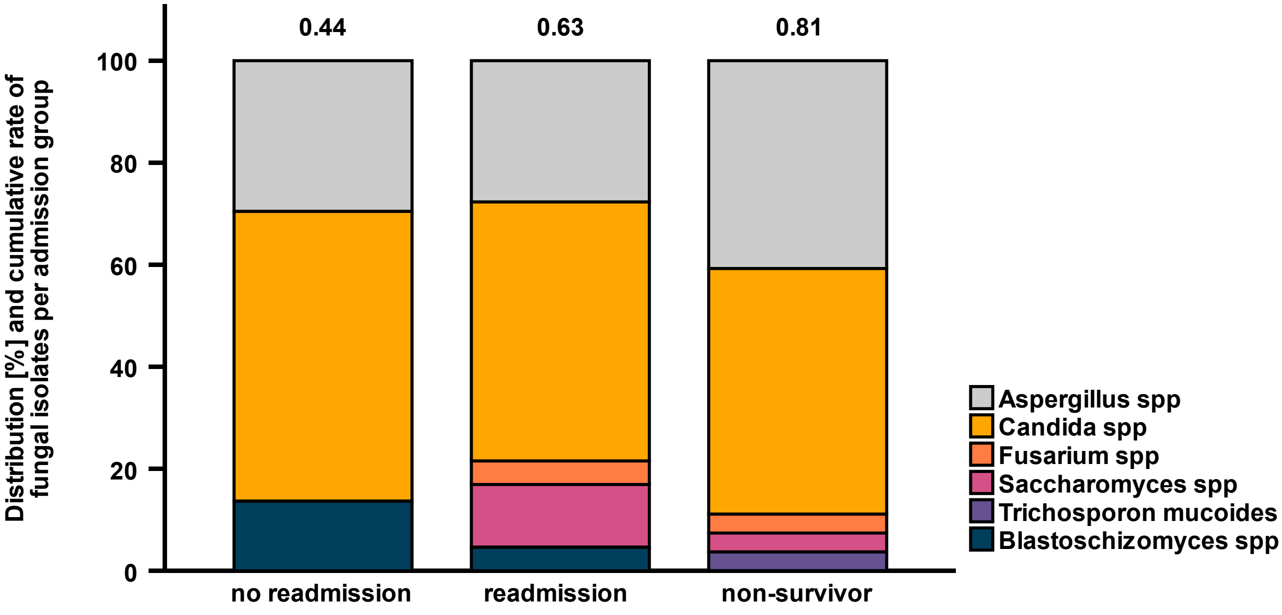
0.44
0.63
0.81
Distribution [%] and cumulative rate of fungal isolates per admission group
100
80
60
40
20
0
no readmission
readmission
non-survivor
Aspergillus spp
Candida spp
Fusarium spp
Saccharomyces spp
Trichosporon mucoides
Blastoschizomyces spp

Additional Figure 2. Receiver operating characteristics (ROC) curves for pSOFA (A) and O-PRISM (B) on every PICU admission. (A)

(A)

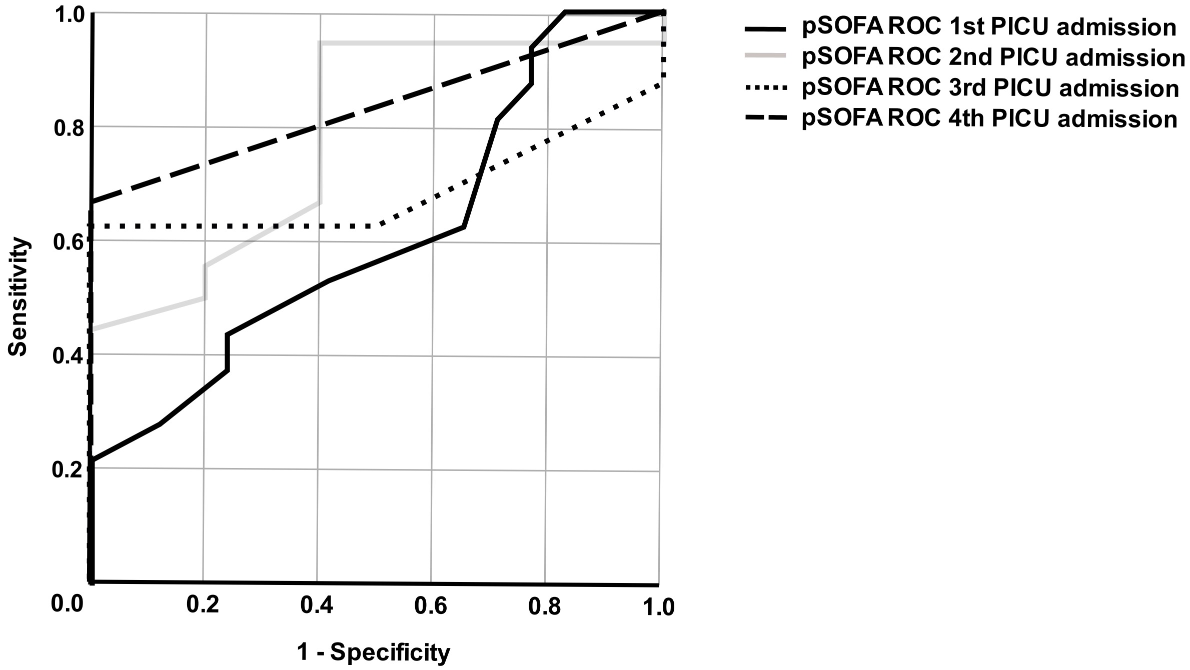

(B)

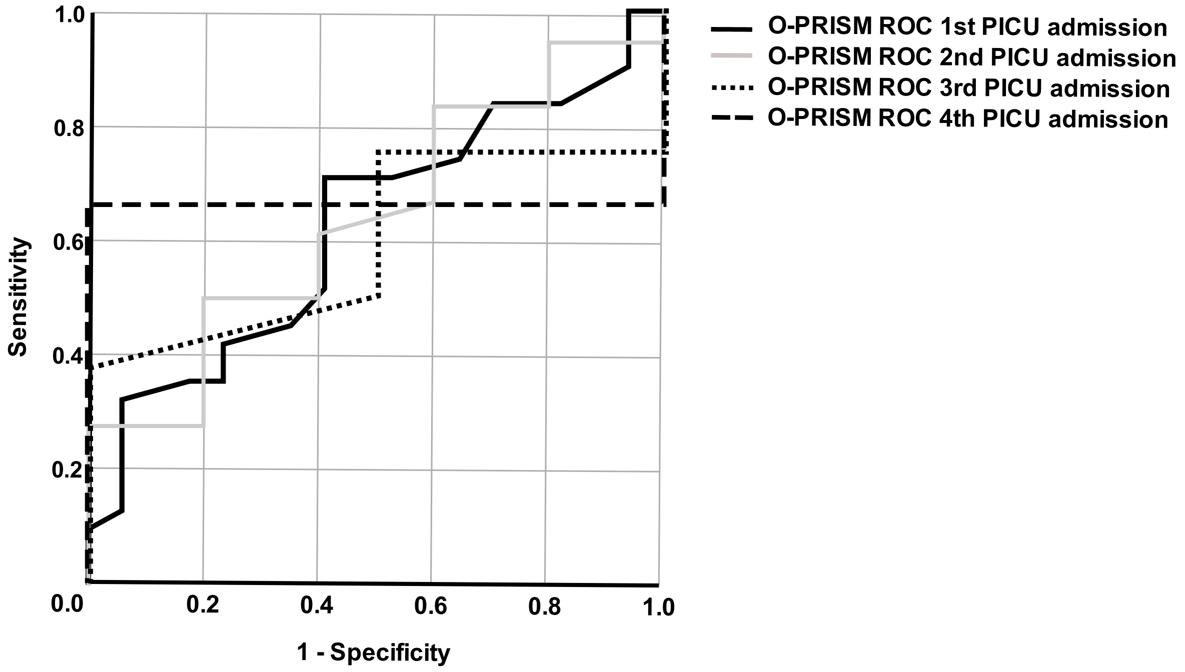

For predictive ability, see Table 4.
